# Mme. Montessori Speaks for Herself

**Published:** 1919-10-25

**Authors:** 


### MME. MONTESSORI SPEAKS FOR HERSELF.

The large lecture-room of the Central Hall, Westminster, was crowded on October 15 for Signora Montessori's public meeting and lecture. A beautiful cushion of flowers was presented by the students attending her training classes, and after a few introductory remarks by Sir George Kekewich, Mme. Montessori spoke in Italian, which was translated, sentence by sentence, very ably, by a young girl. It was interesting to notice that a little ripple of laughter before the translation had been made indicated the presence of a good many people able to understand Italian.

Mme. Montessori spoke of the child as a little stranger in a world entirely disproportioned to its size. Everything in it is made for grown-up people, and while the child can exist it cannot act freely in its environment. How, she asked, should we feel if put in the child's position, living among giants, climbing stairs, using chairs and beds altogether disproportioned to our size, especially if among people who were far more agile and were constantly impatient with our slowness? If eating soup with difficulty, some such impatient being would pounce upon and feed us; if dressing to go out we should be seized and buttoned. The environment is not ready for the child: his work is to grow, and we need to re-educate ourselves to help him to do so.

She emphasised strongly the need of patience with the child's slowness in performing actions which are new to him. If trusted and given time the child of three can lay tables, dress and undress itself. At two and a half she had watched a child carrying a full glass of water, repeating to itself, "Be careful, be careful."

Some colour was certainly given to the idea that "the Montessori system" consists in letting the child do exactly what it pleases, by the story related by the lecturer of a small child whom a nurse came to fetch from her school in Rome. "As I was there the nurse could not do as she wished," for the child insisted upon sitting on the floor to fasten with a button-hook a pair of knee-high gaiters. The nurse suggested that soup at home was getting cold, offered a sweet waiting there, but the child preferred its conquest of difficulty. It must be owned that, however sincere our sympathy with the child, we may doubt if the wheels of life should be made to stand still for its convenience. Certainly in later life they will not do so, and the habit of refusing to leave unfinished work is not unknown, and makes a very tiresome person!

Mme. Montessori went on to speak of the child as a great explorer. *Qualities* attract its attention, to differentiate colour, size, form, sound, surfaces, temperature. And here she notices a remarkable fact. While the child's attention is naturally unstable, wandering butterfly-like from one object to another, the concentration of the busy child is greater than that of the average grown person. A noise in the street, a person entering will scatter our thoughts, so that it is related as a remarkable fact that a certain Italian poet was so lost in thought as not to observe the passing of a noisy wedding procession. She, however, has picked up a chair with a child upon it, set it on a table and allowed forty other children to sing and dance round, yet the child went on undisturbed with its occupation. Success in work she found to be more welcome and satisfying than tangible rewards. On one occasion when she offered a sweet or toy to children playing the "Silence" game of tiptoeing out when called in a whisper, she found that forty played in succession and none wished to stop.

Mme. Montessori's experience is that work with interest does not tire. Children suitably occupied do not react to the tests for fatigue. She pleads for observation, patience, help to learn and to grow, in place of discipline and instruction. Discipline, however, comes through work, the patient surmounting of difficulty, and growth of character.

